# The anterior neck scar outcomes of conventional thyroidectomy using a wound protector: a multicenter double-blinded randomized controlled trial

**DOI:** 10.1097/JS9.0000000000001288

**Published:** 2024-03-18

**Authors:** Woochul Kim, Hyeong Won Yu, Su-jin Kim, Young Jun Chai, June Young Choi, Kyu Eun Lee

**Affiliations:** a Department of Surgery, Seoul National University Hospital, Jongno-gu, Seoul; b Department of Surgery, Seoul National University, Bundang Hospital, Bundang-gu, Seongnam-si, Gyeonggi-do; c Department of Surgery, Seoul National University, College of Medicine, Jongno-gu; d Department of Surgery, Seoul National University, Boramae Medical Center, Seoul, Korea

**Keywords:** general surgery, neck, quality of life, scars, thyroidectomy, wound

## Abstract

**Purpose:**

This study aimed to investigate the effectiveness of a novel wound protector in enhancing the cosmetic outcomes of thyroidectomy.

**Material and methods:**

This multicenter, double-blinded randomized controlled trial enrolled 129 patients undergoing open thyroidectomy. The patients were divided into a wound protector group and a control group. Subjective patient assessments were conducted, measuring wound satisfaction, pain, and itchiness. Additionally, blinded observers evaluated scars using the Vancouver Scar Scale.

**Results:**

The Vancouver Scar Scale revealed significant advantages for the wound protector group, demonstrating improvements in pigmentation (*P*=0.002), vascularity (*P*=0.014), pliability (*P*=0.001), and height (*P*=0.001).

**Conclusion:**

The thyroid wound protector offers a potential to improve postoperative cosmetic outcomes. Further research is warranted to explore patient experiences and optimize the application of this innovative wound protector across diverse surgical contexts.

## Introduction

Highlights

- It is the first multicenter, double-blinded, randomized control trial for wound protector in thyroid surgeries.
- Blinded, trained researchers assessed patients using Vancouver Scar Scale preoperations and postoperations.
- During operation, the wound protector facilitates adequate wound exposure while also providing protective elements.
- Wound protector group demonstrated superior outcomes in pigmentation, vascularity, pliability, height, and overall scores.

Thyroidectomy stands as a demanding surgical procedure for endocrine surgeons, not due to the morbid outcomes of thyroid cancers but rather due to the high expectations of patients. Thyroid cancer patients generally experience longer overall survival than those with other types of cancer. As a result, complications stemming from thyroidectomy can significantly impact patients’ quality of life. Consequently, patients often seek safe surgical approaches with minimal complications that may affect their lifespan. The 2020 Globocan Report reveals that ~600 000 individuals are diagnosed with thyroid cancer each year, with a notable over-representation of young women^1^. This sex disproportion in thyroid cancer incidence has prompted a growing interest in achieving cosmetically superior incisions.

Presently, surgical intervention remains the sole curative treatment option for thyroid cancer, necessitating an incision directly on the anterior neck. The early incisions, originally described by Theodor Kocher, were vertical, but over time, these incisions evolved into horizontal ones. Modern neck incisions for thyroidectomy are typically situated 1–2 cm superior to the sternal notch and range from 4 to 7 cm in length, depending on the surgeon’s surgical preferences^2,3^. Various intraoperative factors can influence the postoperative healing of these incisions. Firstly, excessive retraction force is applied to the skin to enhance visibility of the operative field, potentially compromising adequate blood circulation to the affected skin and leading to excessive scarring postoperatively^4,5^. Secondly, due to the small size of the neck incisions, energy-emitting devices may come into contact with the skin, leading to heat burns that cause edema and render the wound more susceptible to postoperative infections^6,7^. Lastly, the use of intradermal sutures with multifilament threads contributes to excessive inflammation at the wound site^4,8,9^. As there is no better alternative to intradermal sutures, it is imperative for surgeons to minimize other contributing factors to the greatest extent possible.

Currently, there are several wound protectors available for abdominal and thoracic surgery^10,11^, but only a limited number of options for thyroid operations. This discrepancy arises from the perception that wound protectors are primarily designed to prevent contamination. Consequently, many thyroid surgeons tend to omit their use in so-called ‘clean surgery’. Furthermore, some surgeons have raised questions about the efficacy of commercial wound protectors compared to alternatives such as gloves or penrose drains for wound protection. Most of the available products are crafted from soft materials, which, do not significantly add value compared to utilizing already existing methods^12–14^.

**Figure 1:**
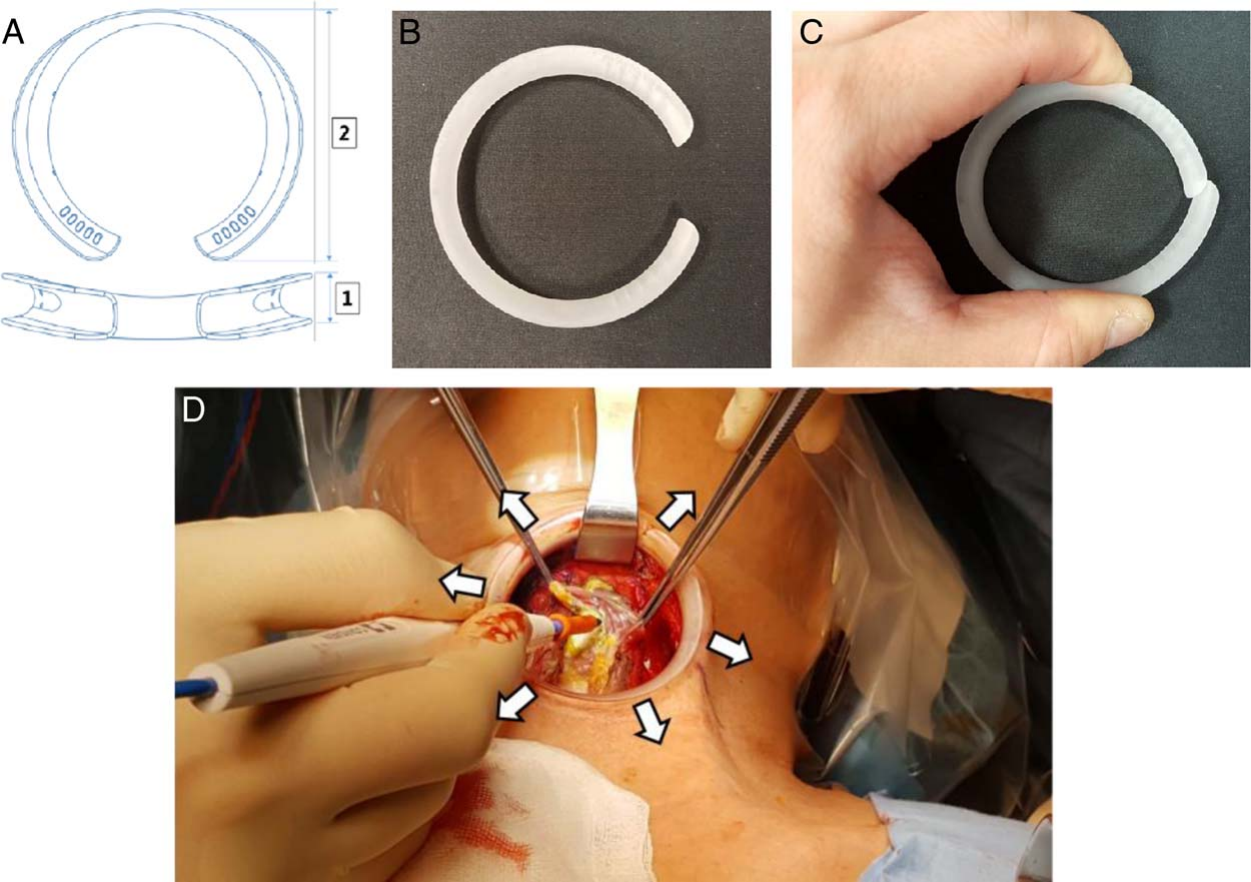
A Illustration of the prototype design of the wound protector design used in this study (C Protector); B When external force is not applied to the protector, recoil force allows for self-retraction; C When compressed, it offers flexibility so that excessive force minimally affects the skin, and it can be easily taken out; D When applied, it facilitates expanded coverage of the operative field.

The conduction of randomized control trials investigating the application of wound protectors in thyroidectomy surgeries is confronted with an array of formidable challenges that extend beyond the expected regulatory, logistical, and collaborative hurdles. The variances in incision size and shape among institutions impede the development of a standardized product sample, while institution-specific wound protection protocols further complicate the establishment of uniform research parameters. The C-protector (SurgiCore) utilized in this study features a distinctive C-shaped design that offers flexibility in adjusting to various incision lengths while its rigidity ensures even distribution of retraction force (Fig. 1). Notably, this protector is designed to break in half, with force directed inwards from each side, allowing for easy removal without the need to stretch the skin. In this study, subjective surveys were administered to patients, and the Vancouver Scar Scale (VSS) served as an objective measurement tool to assess the effectiveness of this wound protector. In light of these multifaceted complexities, this research emerges as a noteworthy exemplar, one that aligns with the stringent criteria necessitated by these intricate factors, to gain valuable insights into how well wound protectors work during thyroidectomy surgeries.

## Methods

### Study design

This study was conducted at two high-volume tertiary hospitals and followed a double-blinded randomized controlled trial design. Patients were informed about the study, and their informed consent was obtained prior to surgery. Institutional Review Board of Seoul National University Hospital and Seoul National University Bundang Hospital, Korea (IRB no. 2010-067-1163, B-2007-624-001) was obtained before the implementation of this study and the study was registered in the Clinical Research Information Service of Korea Disease Control and Prevention Agency (http://cris.nih.go.kr/cris/index/index.do) (CRIS no. KCT0005625).

### Wound protector

The wound protector utilized in this study was the C-protector (SurgiCore, Korea), which was developed and patented by the Seoul National University R&DB Foundation with a unique C-shaped design (Fig. 1). It was constructed from polycarbonate material to serve three primary functions. Firstly, it evenly disperses retraction force across skin incisions when applied. Secondly, the protector featured an opening at one end, facilitating a snug fit to the skin incision, allowing for self-retraction without exerting excessive localized force. Lastly, it shields most of the skin surface from contamination and the heat generated by energy devices.

### Patient selection

**Figure 2:**
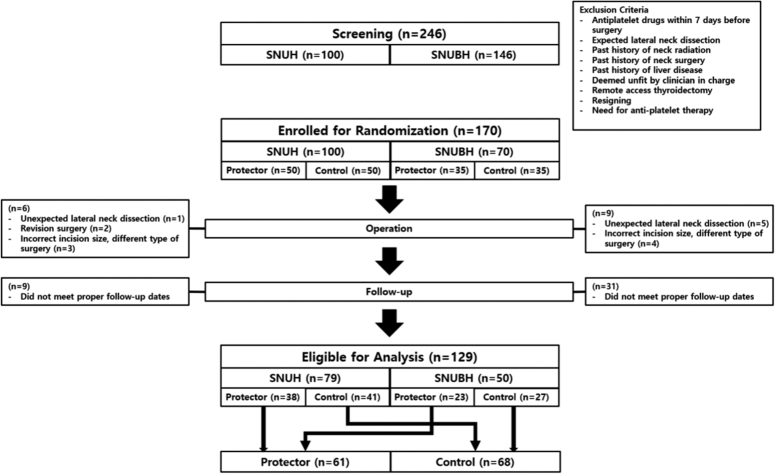
Schematic representation of patient selection process. Patient selection was conducted independently at each hospital. Randomization equally distributed the number of patients to either the protector group or the control group.

The study included patients undergoing open thyroidectomy with incisions ranging from 5 to 6 cm in length. Patients with lateral lymph node metastasis, goiters, and large thyroid nodules requiring extended wound incisions were excluded. Additionally, patients with predisposing factors that could impact wound healing, such as a history of radiation exposure, prior neck surgery, antiplatelet or steroid medication usage, high obesity, irregular hormonal levels, and ages below 20 or above 70, were also excluded. Initially, 246 patients were screened for this study, where 56 patients were ruled out because they fell into the exclusion criteria, or changed to remote-access thyroidectomy. In total, 170 patients were eligible for this study, with 100 patients from Seoul National University Hospital 70 patients from Seoul National University Bundang Hospital. The patient cohort was subjected to a randomized allocation process into distinct groups: the protector group, which received wound protectors, and the control group. This allocation was executed using a random number generator to ensure that the assignment was devoid of bias. The randomization process was conducted in alignment to CONSORT guidelines^15^ (Supplement 1, Supplemental Digital Content 1, http://links.lww.com/JS9/C94, Supplemental Digital Content 2, http://links.lww.com/JS9/C95). After the operation, patients underwent three evaluations: an initial preoperative assessment, a follow-up visit at 2 weeks postoperation, and another visit at three months postoperation. Fifty five patients who were either lost to follow-up or did not meet the required revisit dates were excluded from the study. In total, 129 patients (68 in the control group and 61 in the wound protector group) were evaluated (Fig. 2).

### Surgical procedures

The surgeries were conducted by four highly experienced surgeons, each performing over 200 thyroid surgeries annually. Before the trial, they made 5–6 cm incisions on the anterior neck, ~1–1.5 cm above the clavicle line. A skin flap was created for both the control and wound protector groups. Wound protector was applied to the protector group after the skin flap was created, while the control group had exposed skin throughout the surgery. Prophylactic first-line cephalosporin was administered before the skin incision. The midline closure of strap muscles was consistently performed using vicryl sutures, and hemostatic and antiadhesive agents were applied to the operative bed before wound closure. Wound protector was removed just before platysma muscle closure, and both groups had their skin closed using 5-0 vicryl sutures.

### Postoperative care for scars

After the operation, patients were advised to perform stretching exercises to reduce adhesions. The exercises include bowing, tilting, and rotating the neck, as well as shrugging and stretching the shoulders. However, stretching the neck backward, which could exert excessive force on the scars, is not recommended. After 2 weeks, patients were provided with a 100 ml spray form of polysiloxanes and silicon dioxide from KELO-COTE (Alliance Pharma, UK).

### Outcome measures

**Figure 3:**
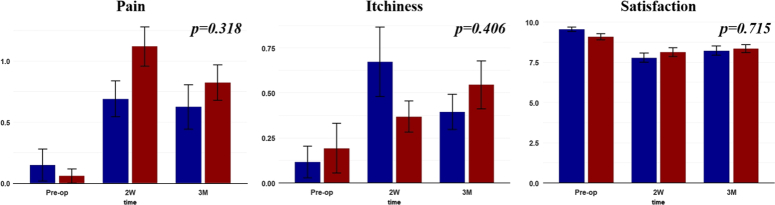
Each bar graph represents changes in the survey scores of each item by time. The protector group is represented by a blue bar (n=61), while the control group is indicated by a red bar (n=66). SD is represented by error bars. ‘Pre-op’ signifies surveys conducted pre-operation, ‘2W’ denotes surveys at 2 weeks postoperation, and ‘3M’ indicates surveys conducted at 3 months postoperation.

In this study, patients completed a subjective survey at each visit. The survey included questions related to postoperative wound satisfaction, pain, and itchiness, rated on a scale from 0 to 10, with 10 indicating the most severe symptoms. For objective analysis, the VSS scores, comprising four items (pigmentation, vascularity, pliability, and height), was assessed by three blinded observers (Fig. 3). Scores from each observer were combined and averaged for analysis.

### Sample size estimation

Sample size was determined considering the expected VSS scores, which was our primary outcome measures. Based on previous literature, mean value of 4.0 with SD of 2.1 was placed for the protector group, and mean value of 5.7 with SD of 2.3 was placed for the control group. Using a two-sided test with 80% power and a significance level of 5%, 23 patients were determined to be in each groups. The study was conducted during COVID-19 pandemic, therefore we expected follow-up loss rate to be 60% where 58 patients were determined to be included in each of the two groups.

### Statistical methods

Patient characteristics between the protector and control groups were compared and analyzed using Student’s *T*-test and *χ*^2^ statistical analysis. The linear mixed model was used for the analysis of subjective survey scores and the VSS scores. Rstudio version 1.2 (Posit, Boston, US) software was used for randomization and SPSS 27.0 (IBM) software was used for statistical analysis.

### Theory/Calculation

The wound protector is designed to serve a dual purpose, safeguarding the skin from unwarranted heat and potential contamination. Additionally, its functionality includes the facilitation of wound retraction, thereby mitigating the need for manual retractions during surgical procedures. This dual mechanism is anticipated to contribute to a reduction in inflammation within the peri-wound tissues. The consequential decrease in inflammation is expected to translate into alleviated pain and itchiness symptoms for patients, ultimately fostering enhanced overall satisfaction. It is also postulated that the employment of the wound protector will yield improvements in the VSS scores. This conjecture is grounded in the premise that the protective measures afforded by the device would collectively foster a more favorable healing environment, leading to superior cosmetic outcomes.

To substantiate these hypotheses, the linear mixed model analysis is poised to discern potential differences in outcomes between the control group and the protector group. The protector group should show significant statistical differences, with having overall superior outcomes.

## Results

### Patient characteristics

A total of 129 patients were included in this study, with 61 patients allocated to the protector group and 68 patients to the control group. The majority of enrolled patients from both the protector group and the control group were female (77.1 vs. 77.9%) with mean ages of 47 and 53, respectively. The youngest patient participating in this study was 27 years old, and the oldest was 69 years old. The extent of surgery was, in general, limited to thyroid lobectomy for both groups. The average operation time took ~75 min, and on average, patients were discharged 2 days postoperatively. Distribution of possible confounding factors were evaluated, where there were no significant statistical differences for sex, age, BMI, diabetes, hypertension, dyslipidemia, extent of surgery, and operation time. Detailed analysis is summarized in Table 1.

**Table 1:** Baseline characteristics of patients in each group.

	Protector (*n*=61)	Control (*n*=68)	*P*
Sex			0.904
Male	14 (22.9%)	15 (22.1%)	
Female	47 (77.1%)	53 (77.9%)	
Age	51 (range : 28–69)	49 (range: 27–69)	0.346
BMI (kg/m^2^)	25.52 (range: 18.1–41.3)	25.68 (range: 17.0–39.6)	0.922
Smoking	11 (18.0%)	11 (16.2%)	0.779
Diabetes	5 (8.2%)	7 (10.3%)	0.682
Hypertension	15 (24.6%)	15 (22.5%)	0.734
Dyslipidemia	9 (14.8%)	9 (13.2%)	0.804
Operation			
Isthmectomy	2 (3.3%)	2 (2.9%)	
lobectomy	47 (77.0%)	49 (72.1%)	
total thyroidectomy	12 (19.7%)	17 (25.0%)	
Operation time (min)	75.7 (range: 35–125)	74.4 (range: 30–145)	0.801
Date to discharge (days)	2.23 (range: 2–3)	2.26 (range: 1–5)	0.695

### Subjective patient assessment

The patient survey included inquiries related to pain, itchiness, and overall satisfaction with the surgical scar on scales of 10. At both 2 weeks and three months after the operation, the mean survey scores regarding pain were lower in the protector group compared to the control group. The mean scores were 0.688 and 0.622 for the protector group at 2 weeks and 3 months, respectively, while it was 1.117 and 0.826 for the control group. Although differences in the values were observed, no statistical difference was observed regarding pain symptoms (*P*=0.318). Regarding itchiness symptoms, the protector group showed higher scores at two weeks postoperation with 0.672 while the control group showed 0.368. However, at 3 months postoperation, the scores were lower in the protector group with 0.393, with the control group having 0.544. No significant statistical difference was observed in the itchiness symptom as well (*P*=0.406). Despite the overall trend of fewer symptoms in the protector group compared to the control group, mean scores for overall scar satisfaction were higher in the control group. At 2 weeks, the protector group had a mean score of 7.770, while the control group had 8.118. Moreover, at three months, the protector group had 8.213, while the control group showed 8.338. Although no statistical differences were seen between the two groups (*P*=0.715), the protector group showed a steeper score drop at 2 weeks compared to pre-operation scores. The detailed results are indicated in Figure 3.

### Vancouver scar scale assessment by blinded observers

**Figure 4:**
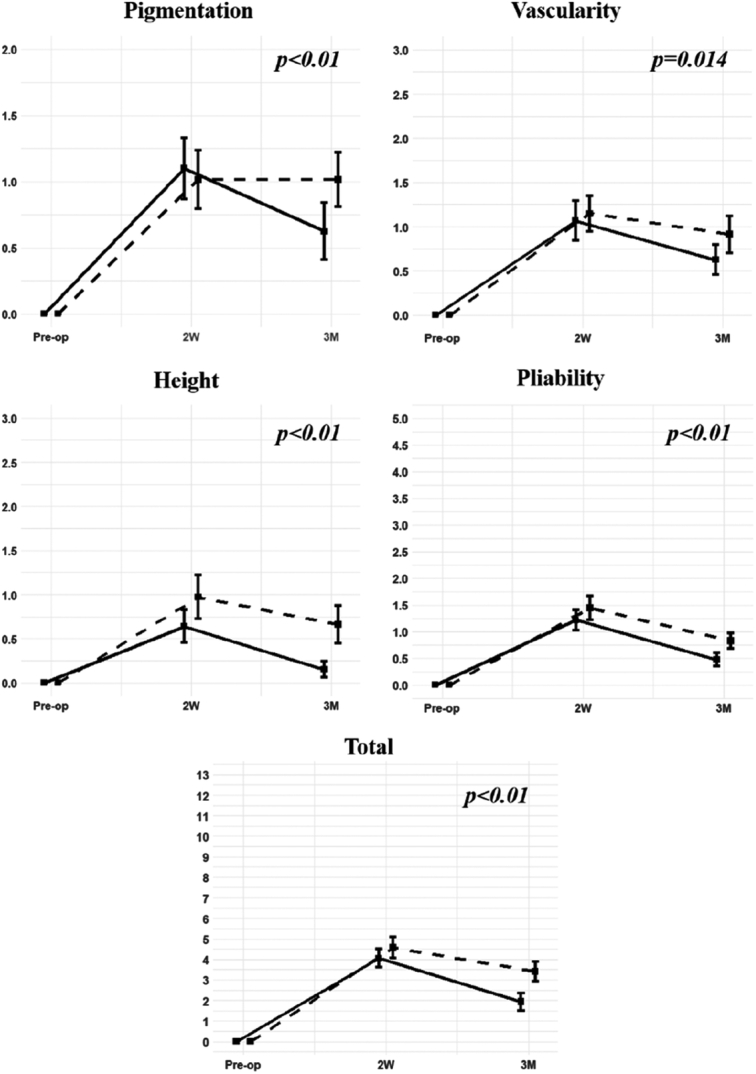
The lines within each box illustrate changes in scores for each item on the Vancouver Scar Scale. Solid lines correspond to the protector group (*n*=61), while dashed lines signify the control group (*n*=68). SDs are depicted as error bars.

Objective assessments were conducted using the Vancouver Scar Scale, encompassing four categories (pigmentation, vascularity, pliability, and height), culminating in an overall score (Fig. 4). Blinded observers examined patients’ necks preoperatively and postoperatively, with the initial preoperative examination set as a reference value for the upcoming postoperative examinations. For pigmentation, the protector group scored 1.131 at 2 weeks, higher than the control group’s score of 1.015. However, at 3 months, the scores reversed, with the protector group at 0.623 and the control group at 1.015, showing significant statistical differences in changes (*P*=0.002). Vascularity scores differed, with the protector group scoring 1.066 at 2 weeks compared to the control group’s 1.147. At 3 months, the protector group showed 0.623, while the control group had 0.912, demonstrating statistical differences in changes (*P*=0.014). Similar trends were seen for pliability scores at 2 weeks (protector: 1.213, control: 1.441) and 3 months (protector: 0.475, control: 0.824), with significant differences in changes (*P*=0.001). For height scores, the protector group scored 0.639 at two weeks, and the control group scored 0.971. At 3 months, both groups had declined values (protector: 0.213, control: 0.662), showing significant statistical differences in changes (*P*=0.001). Total scores for the Vancouver Scar Scales also exhibited significant statistical differences between the protector and control groups (*P*=0.001).

## Discussion

Surgical interventions for thyroid and parathyroid diseases often culminate in a conspicuous 4–6 cm linear scar on the anterior neck, thereby underscoring the increasing significance of cosmetic outcomes. Notably, previous studies have reported that neck scars can substantially impact overall quality of life, irrespective of scar type^5,16^. Concern for scars is added when neck scars become keloid, where scars become more prominent as they become redder than surrounding tissues, becoming more conspicuous. A primary concern frequently expressed by patients during clinical consultations pertains to neck scars, encompassing issues such as adhesions and cosmetic outcomes. Many patients have articulated a preference for scars in less visible bodily regions^17^. Although numerous commercial wound protectors have been introduced with the intention of ameliorating scar outcomes, their widespread acceptance and utilization remain constrained. It is pivotal to acknowledge that the existing body of research pertaining to these products is relatively limited in scope. Nevertheless, the available studies have shown encouraging results within specific contexts, underscoring the potential for favorable cosmetic outcomes^12–14^.

Numerous techniques are available to mitigate scarring in surgical procedures, with the most prevalent approach being the utilization of wound protection devices. While certain articles have explored alternative methods involving readily available materials within the operating theater, such as penrose drains or powder-free surgical gloves^18,19^, these cost-effective options necessitate attachment to the open incision surfaces, resulting in minor suture marks post-surgery. In contrast, commercialized wound protection devices offer a more seamless and scar-minimizing application, albeit at an additional cost. Some surgeons have even ventured into injecting botulinum toxin directly into incisions, demonstrating superior short-term cosmetic outcomes when compared to normal saline injections^20^. Furthermore, the introduction of dermal staplers, a technique commonly employed in breast surgery, has exhibited promising long-term cosmetic benefits when applied to thyroidectomy patients^21^. Nevertheless, these diverse methods have not been conducted on larger number of patients.

This study represents a comprehensive investigation into the effectiveness of a novel wound protector, and it stands out as one of the largest studies conducted to date in this field. The research was meticulously designed as a multicenter, double-blinded randomized controlled trial, specifically aimed at mitigating selection bias. Objective patient evaluations were carried out by impartial, blinded observers using the VSS scores at the outpatient clinic, with no knowledge of which patient belonged to a particular group. The VSS scores revealed remarkably improved outcomes in various critical parameters, including postoperative wound pigmentation, vascularity, pliability, and height. These results collectively point to an overall lower VSS scores for patients who benefited from the novel wound protector.

However, it is important to note that the initial intention behind developing the VSS scoring system was to assess scars resulting from burn wounds. Despite the original purpose of the VSS scores, the rationale for choosing the VSS scoring system over other modalities, such as the Patient and Observer Scar Assessment Scale (POSAS), is that VSS has been more frequently utilized in the past and is still widely implemented in various scar studies^22–24^. Another intention was to establish a reference value of VSS scores for future studies involving anterior neck scars. To address the lack of patient assessment in the VSS system, we included a subjective symptom survey in this study. Additionally, it is advisable to interpret the outcome VSS scores in this study with caution. For instance, score of 1 in the pigmentation parameter indicates hypopigmentation, whereas 2 indicates hyperpigmentation. Hypopigmentation may not necessarily relate to worse scar outcomes compared to hyperpigmentation. However, in a study composing of mostly light skinned ethnically Korean population, hypopigmentation may relate to better aesthetic outcomes compared to hyperpigmentation. Therefore, in our study, lower pigmentation scores does reflect better aesthetic scar outcomes, but it cannot be universally applied to all groups of patients. Moreover, changes in the VSS scores may not directly reflect physiologic wound healing process. Theoretically, since fibrosis occurs actively starting from 2 weeks, height scores should be higher in the three months period compared to 2 week period. It was shown in this study that scores for height decreased at three months. There can be multiple factors that may influence height of the scar, including level of fibrosis, localized bleeding from capillaries, and serous secretions. VSS scores revealed higher scores for pigmentation, vascularity, and pliability at 2 weeks compared to three months period. All the aforementioned factors, with addition of the effect of scar topical spray applied in this study (KELO-COTE) may have been attributable to observation of higher elevated scars. Therefore, further studies utilizing VSS scoring should interpret the outcomes with context of their study design rather than making direct conclusions based on VSS scores alone.

The overall lower VSS scores seen in the protector group can be attributed to several inherent attributes of its design. First and foremost, it effectively guards against potential burn injuries that can occur from energy devices or bovie tips coming into contact with the skin edges of the surgical wound. Moreover, it serves as a protective barrier for subcutaneous tissue, preserving vital blood flow and minimizing the risk of hemorrhage. The rigidity of the wound protector plays a pivotal role in maintaining wound openness during its application, reducing the need for excessive skin traction and ultimately enhancing the wound healing process. As a result, patients in the wound protector group exhibited white, linear scars with minimal redness, in stark contrast to the elevated and more prominent scar healing observed in the control group, as indicated in Figure 3.

The surgical application of the novel wound protector used in this study was feasible, yet it still had boundaries. During our initial trials with the wound protector before conducting the study, there were instances where the incision was too large for the protector to fit snugly. As a result, the wound protector would occasionally fall off during the operation, requiring the operator to re-apply it. However, as long as the size of the incision is consistently uniform, the wound protector offers substantial advantages for surgeons during the intraoperative experience. It facilitates exposure of the operative field without the need for excessive retraction, thus enabling surgeons to perform procedures with minimal assistance. This is particularly valuable for surgical teams, as it allows for the efficient utilization of energy devices at various angles, as the wound protector covers the skin and underlying subcutaneous tissues. These features may empower surgeons to create shorter scars than usual, ultimately leading to superior cosmetic outcomes for patients and enhancing the overall surgical experience.

Nonetheless, this study is not without its limitations. Firstly, it is subject to bias, given that the surgeon can discern which patients are using the wound protector. Furthermore, the standardization of surgical procedures, although indispensable for securing consistency and result reliability, may not fully account for variations in surgical styles or techniques. Consequently, careful consideration is warranted, recognizing that the findings in this study may not be universally generalizable across all surgical contexts.

In summary, the novel wound protector emerges as a valuable addition to the armamentarium of tools available for thyroid and parathyroid surgeries, demonstrating its potential to enhance postoperative cosmetic outcomes. Subsequent research endeavors should aim to delve into the subjective patient experiences and further refine the application of this innovative wound protector across diverse surgical settings.

## Ethical approval

The standard care for thyroid surgery permits both utilizing and not utilizing a wound protector. Therefore this research study did not require additional ethical approval.

## Consent

Written informed consent was obtained from the patient for publication of this case report and accompanying images. A copy of the written consent is available for review by the Editor-in-Chief of this journal on request.

## Sources of funding

This work was supported by the Korea Medical Device Development Fund grant funded by the Korea government (the Ministry of Science and ICT, the Ministry of Trade, Industry and Energy, the Ministry of Health & Welfare, the Ministry of Food and Drug Safety) (Project Number: RS-2020-KD000244).

## Author contribution

W.K.: writing – original draft, writing – review and editing, and formal analysis; H.W.Y.: conceptualization, writing – original draft, and writing – review and editing; –S.-J.K.: investigation and methodology; –Y.J.C.: investigation and methodology; –J.Y.C.: conceptualization and supervision; –K.E.L.: conceptualization, supervision, writing – review and editing, and project administration.

## Conflicts of interest disclosure

The author declares no conflicts of interest.

## Research registration unique identifying number (UIN)

1. Name of the registry: Clinical Research information Service (www.cris.nih.go.kr).
2. Unique identifying number or registration ID: KCT0005625.
3. Hyperlink to your specific registration (must be publicly accessible and will be checked): https://cris.nih.go.kr/cris/search/detailSearch.do?seq=17634.

## Guarantor

Hyeong Won Yu.

## Data availability statement

The data that support these findings are available upon request from the corresponding author.

## Provenance and peer review

Not commissioned, externally peer-reviewed.

## Presentation

None.

## Additional information

Correspondence and requests for materials should be addressed to H.W.Y. and K.E.L.

## Supplementary Material
