# Supplementary figures and images for: The anterior neck scar outcomes of conventional thyroidectomy using a wound protector: a multicenter double-blinded randomized controlled trial
Source: Int J Surg. 2024 Mar 18;110(6):3425–32. doi: 10.1097/JS9.0000000000001288 (PMC11175810; doi:10.1097/JS9.0000000000001288)

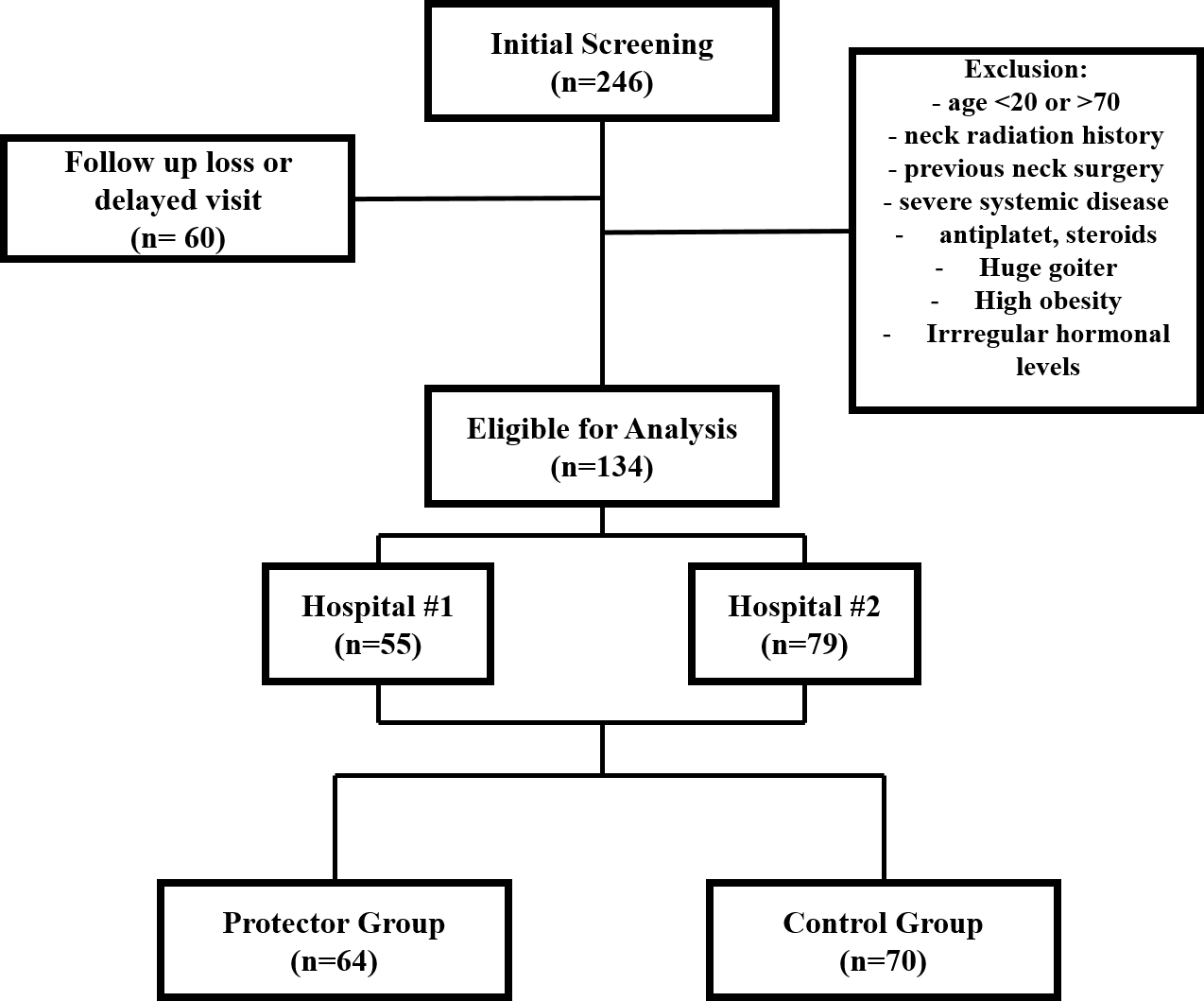

Supplement: Supplementary file 2 [file js9-110-3425-s002.docx]
